# Frequency and Circumstances of Falls Events in People Living With Spinal and Bulbar Muscular Atrophy: A Cross‐Sectional Survey

**DOI:** 10.1002/pri.70342

**Published:** 2026-09-07

**Authors:** Laurence E. Lee, Luca Zampedri, Dipa Jayaseelan, Carlo Rinaldi, Pietro Fratta, Gita M. Ramdharry

**Affiliations:** ^1^ UCL Queen Square Institute of Neurology University College London London UK; ^2^ Queen Square Centre for Neuromuscular Diseases University College London Hospitals NHS Foundation Trust London UK; ^3^ Department of Paediatrics University of Oxford Oxford UK

## Abstract

**Background and Purpose:**

Spinal and bulbar muscular atrophy (SBMA) is an adult‐onset X‐linked neuromuscular disorder associated with progressive weakness, sensory involvement and impaired mobility. Falls appear frequent in SBMA, but their real‐world frequency and circumstances have not been systematically described. This study investigated the frequency, context and perceived causes of falls and near‐falls in adults with SBMA.

**Methods:**

A cross‐sectional survey was conducted in adults with genetically confirmed SBMA attending a UK specialist clinic. Participants completed a modified Falls Events Questionnaire, reporting fall and near‐fall frequency over the previous 12 months and describing up to three events of each type. Participant‐level analyses described prevalence and frequency, while event‐level analyses descriptively summarised circumstances.

**Results:**

Of 83 questionnaires distributed, 61 were returned (73.5%). 55 participants (90%) reported at least 1 fall and 42 (69%) reported falls at least monthly; 51 (84%) reported at least one near‐fall. The 55 fallers described 153 fall events (up to three per participant). At event level, most occurred indoors (58%) and during walking, reaching/bending or turning. Participants most commonly attributed falls to muscle weakness (44%), tripping (18%) and loss of balance (17%). Bruising and sprain/strain were common, while fractures were rare (2%). The 51 participants reporting near‐falls described 153 events; walking was the most common activity at the time of the near‐fall, and grabbing a stable surface was the most commonly reported strategy used to avert a fall.

**Discussion:**

Falls and near‐falls were frequently reported in this SBMA cohort and commonly occurred during everyday mobility tasks. Participants most often attributed events to weakness, loss of balance and tripping. These descriptive findings support greater clinical attention to falls in SBMA and may inform assessment and rehabilitation priorities, while prospective studies are needed to establish mechanisms and intervention effectiveness.

## 1 Introduction

Falls are a leading cause of unintentional injury and mortality worldwide (World Health Organization 2021). In 2020 alone, accidental falls accounted for over 6400 deaths in England and Wales (NHS Digital 2022). Beyond physical injury, falls can contribute to fear of falling, reduced mobility, loss of independence and hospitalisation (Vaishya and Vaish 2020). In people with chronic or progressive neurological conditions, these risks may be amplified due to impaired balance (Allen et al. 2013), muscle weakness (Berends et al. 2019), and sensory deficits (Reeves et al. 2021), yet the mechanisms and consequences of falls remain under‐explored in many rare neuromuscular disorders.

Spinal and bulbar muscular atrophy (SBMA), also known as Kennedy's Disease, is a rare, adult‐onset, X‐linked neuromuscular disorder caused by an expanded CAG repeat in the androgen receptor gene (La Spada et al. 1991). Although traditionally estimated to affect around 1 in 30,000 men, recent evidence suggests prevalence may be substantially higher (Andrew et al. 1997; Zanovello et al. 2023). SBMA causes slowly progressive bulbar and limb weakness, with muscle cramps, fasciculations, tremor, fatigue and sensory neuropathy also common (La Spada et al. 1991; Fratta et al. 2014). Despite its slow progression, the cumulative impact on mobility and postural stability is substantial (Alqahtani et al. 2023; Anagnostou et al. 2019). Longitudinal cohort studies have shown that the median age of walking aid use is around 59 years, with a high proportion of ambulatory individuals reporting difficulty with walking, fatigue, and frequent falls (Atsuta et al. 2006; Fernández‐Rhodes et al. 2011). In one cohort, over 80% reported problems with falling and two‐thirds experienced at least one fall over 2 years (Fernández‐Rhodes et al. 2011).

In the general population, the most well‐established fall risk factors include muscle weakness, impaired balance, prior history of falls, use of gait aids, and sensory deficits (Appeadu and Bordoni 2026). Individuals living with SBMA frequently present with all five, and their balance impairments may be further complicated by impaired proprioception secondary to sensory neuropathy. Falls in SBMA have only recently received focussed investigation. Emerging evidence suggests balance impairment may involve static and dynamic control deficits, impaired postural reactions and altered lower‐limb strength (Shrader, Sansare, Niemic, et al. 2025), while measures of mobility, ankle plantar‐flexion strength and endurance may help identify ambulatory men at increased fall risk (Shrader, Niemic, et al. 2025). However, these clinical studies provide limited information about how falls and near‐falls occur in everyday life.

Retrospective surveys in neuromuscular conditions with overlapping motor and sensory impairment, including Charcot–Marie–Tooth disease (CMT), have identified frequent indoor falls, trips during walking and participant‐perceived contributions from lower‐limb weakness (Ramdharry et al. 2018; Hiscock et al. 2014). Equivalent real‐world falls‐event data are lacking in SBMA.

This cross‐sectional survey therefore aimed to determine the frequency and context of falls and near‐falls in adults with genetically confirmed SBMA, and to describe the circumstances and perceived causes of these events to better characterise fall risk in daily life.

## 2 Method

### 2.1 Study Design and Participants

We conducted a cross‐sectional survey of adults with genetically confirmed spinal and bulbar muscular atrophy (SBMA) attending a UK specialist clinic. Eligibility criteria were: age ≥ 18 years, confirmed SBMA, ambulant or previously ambulant, and ability to complete the survey in English. Individuals without genetic confirmation or unable to self‐complete were excluded. No formal cognitive or memory screening was undertaken as part of the study. Ethical approval was obtained from the North West‐Haydock Research Ethics committee, REC reference 24/NW/0218. All participants provided informed consent.

### 2.2 Survey Tool

Data were collected using a modified version of the Falls Events Questionnaire (FEQ), originally developed for use in neurological conditions (Ashburn et al. 2008). The FEQ asks individuals to report on the circumstances surrounding up to three falls and three near‐falls within the past 12 months. It has been previously adapted for self‐completion and successfully used in other neuromuscular populations (Hiscock et al. 2014).

For those who reported falls, detailed questions captured data on activity at the time of the fall, location, perceived cause, preventative strategies, and consequences such as injuries or emotional responses. Similar items were included for near‐fall events (Table 1). A fall was defined for respondents as ‘an event resulting in coming to rest on the ground or a lower level’, and a near‐fall as ‘a loss of balance that would have resulted in a fall without recovery’. Participants also reported their age and estimated falls frequency using predefined response categories. Falls and near‐falls were retrospectively self‐reported. No external sources were used to verify reported events or their circumstances.

**TABLE 1:** Questions on the circumstances of falls and near falls from the falls events questionnaire (Ashburn et al. 2008).

For the last three falls that you remember, try to recall the following information	Where were you when you fell? What were you doing or trying to do at the time? What do you think caused you to fall? Do you remember how you landed? Were you injured?
For the last three near falls that you remember, try to recall the following information	What sort of things were you doing when you nearly fell? Why do you think you nearly fell? How did you save yourself from falling?

*Note:* Open text boxes were available for text in the administered version.

Free‐text responses describing the circumstances and perceived causes of falls and near‐falls were coded at event level into predefined categories informed by the Falls Events Questionnaire response domains and previous falls‐event surveys. Responses describing ‘legs giving way’, ‘knees buckling’, ‘leg weakness’ or similar wording were coded as muscle weakness. Responses describing catching the foot, stumbling, or tripping were coded as tripping, unless the participant specifically attributed the event to uneven or slippery ground, in which case the response was coded as environmental surface. General descriptions such as ‘lost balance’ or ‘overbalanced’, without a more specific stated cause, were coded as loss of balance. Where participants described more than one possible cause, the cause most clearly emphasised by the participant was coded as the primary perceived cause. Initial coding was undertaken by the lead author, with ambiguous responses reviewed with a second researcher and resolved by consensus. Formal inter‐rater agreement was not calculated.

The survey was administered either electronically, in clinic visits, or by post, depending on participant preference. The amended questionnaire and study protocol were reviewed by patient representatives for clarity and accessibility. A 4‐month window was allowed for responses.

### 2.3 Data Analysis

Data were anonymised at entry. Descriptive summaries were generated in Microsoft Excel. Participant‐level outcomes (e.g., prevalence of falls/near‐falls, fall‐frequency categories) used *n* = 61 as the denominator. Event‐level summaries of circumstances (activity, location, perceived cause, injury, prevention) used completed responses only, with denominators reported in the Results (falls *n* = 153 events; near‐falls *n* = 153 events). As participants could contribute up to three events of each type, event‐level observations represented repeated reports within participants and were summarised descriptively rather than treated as independent observations for inferential analysis. Falls frequency was categorised a priori (at least once/day, week, month, in 6 months, year); frequent fallers were defined as ≥ monthly (including weekly/daily). Exploratory differences in age between fallers and non‐fallers were examined using Welch's *t*‐test (unequal variances). Associations between age and ordinal fall‐frequency were assessed using Kendall's *τ* and Spearman's *ρ*. Comparisons of injury proportions between frequent and infrequent fallers were presented descriptively due to small expected cell counts. All tests were two‐sided with *α* = 0.05; analyses were exploratory and no multiplicity adjustments were applied. Coding procedures for free‐text event descriptions are described in Section 2.2. Missing data were handled by available‐case analysis; no imputation was performed.

## 3 Results

Of the 83 questionnaires distributed, 61 were returned (73.5%). Participants had a mean age of 59.9 years (SD 10.7); the median was 59 years (IQR 53.0–69.5), with a range of 37–79 years, and 21/61 (34.4%) were aged ≥ 65. Six participants (9.8%) reported no falls in the previous 12 months, while 55/61 (90.2%) reported at least one fall. These prevalence and frequency findings are participant‐level outcomes. Among the 55 fallers, each participant could describe up to three fall events (potential *n* = 165); 153 fall events were described across all circumstance domains. The event‐level analyses below therefore represent repeated events contributed by individual participants and are descriptive rather than independent observations.

### 3.1 Fall Frequency

**FIGURE 1:**
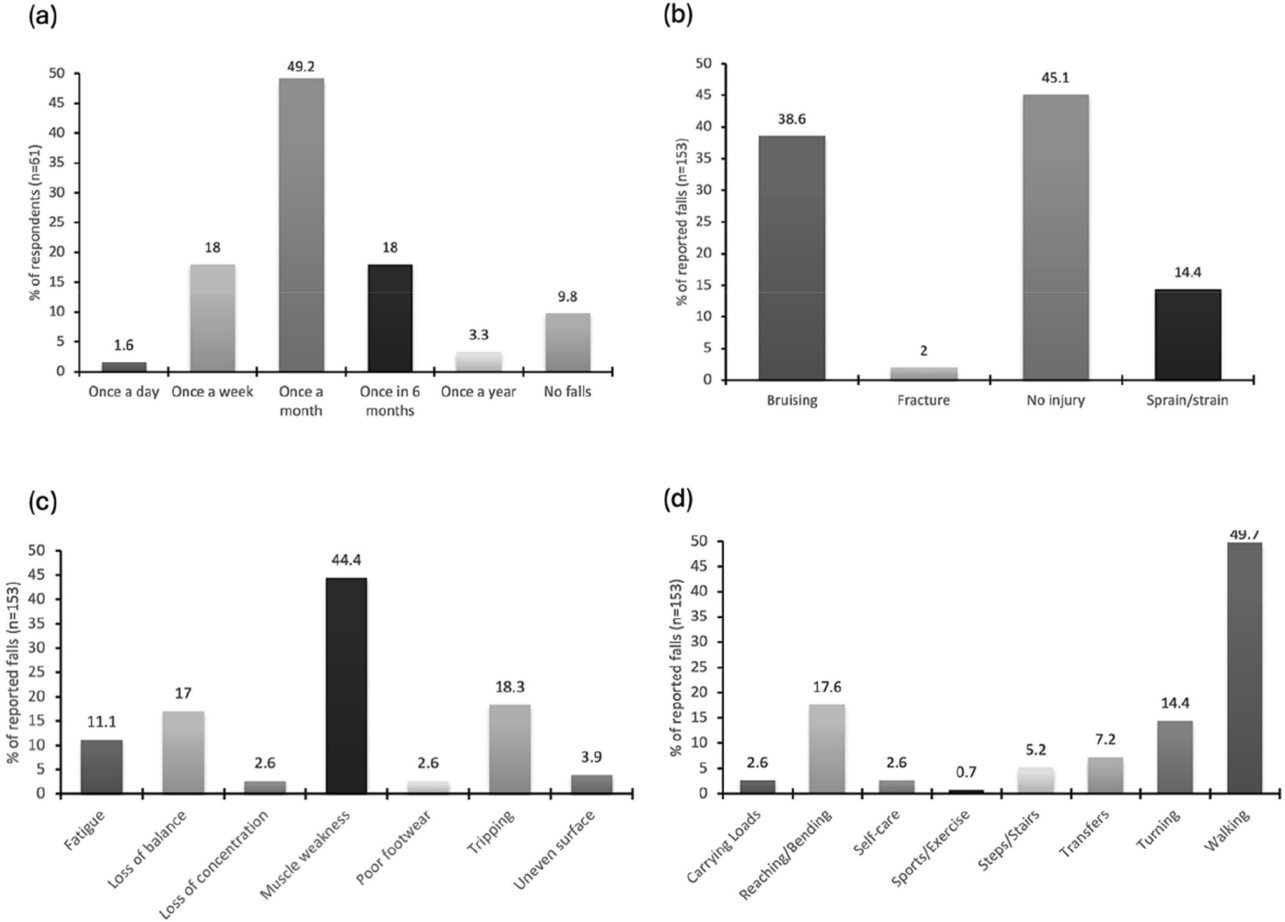
(a) Participant‐reported frequency of falls in the previous 12 months (participant level; *n* = 61); (b) injuries sustained, (c) perceived causes of falls and (d) activities at the time of falling across 153 reported fall events contributed by 55 participants (event level; up to three events per participant).

Across the full cohort (*n* = 61), reported fall frequency was at least once a day in 1.6%, at least once a week in 18.0%, at least once a month in 49.2%, at least once in 6 months in 18.0%, and about once a year in 3.3%. Defining frequent fallers as those reporting falls at least monthly, 42/61 (68.9%) met this criterion (Figure 1). Age showed weak, non‐significant associations with fall frequency (Kendall's *τ* = 0.11, *p* = 0.31; Spearman's *ρ* = 0.14, *p* = 0.30).

### 3.2 Circumstances of Falls

At the event level, among the 153 reported fall events contributed by 55 participants, the majority of falls occurred indoors (89/153, 58.2%), with the remainder outdoors (64/153, 41.8%). Events most often happened during walking—accounting for 32.7% of all described falls indoors and 17.0% outdoors—followed by reaching or bending (17.6%) and turning (14.4%). Smaller proportions were reported during transfers (7.2%), on steps or stairs (5.2%), while carrying loads (2.6%), during self‐care (2.6%), or during sports/exercise (0.7%). Participants most frequently attributed falls to muscle weakness (44.4% of events), tripping (18.3%), general loss of balance (17.0%) and fatigue (11.1%), with fewer reports of uneven surfaces (3.9%), poor footwear (2.6%) and loss of concentration (2.6%). These categories represent participants' perceived causes rather than objectively measured biomechanical mechanisms. Injury details were provided for 153 events; no injury was reported in 45.1%, while bruising occurred in 38.6% and sprain/strain in 14.4%. Fractures were reported in 2.0% of falls.

### 3.3 Near‐Fall Frequency

**FIGURE 2:**
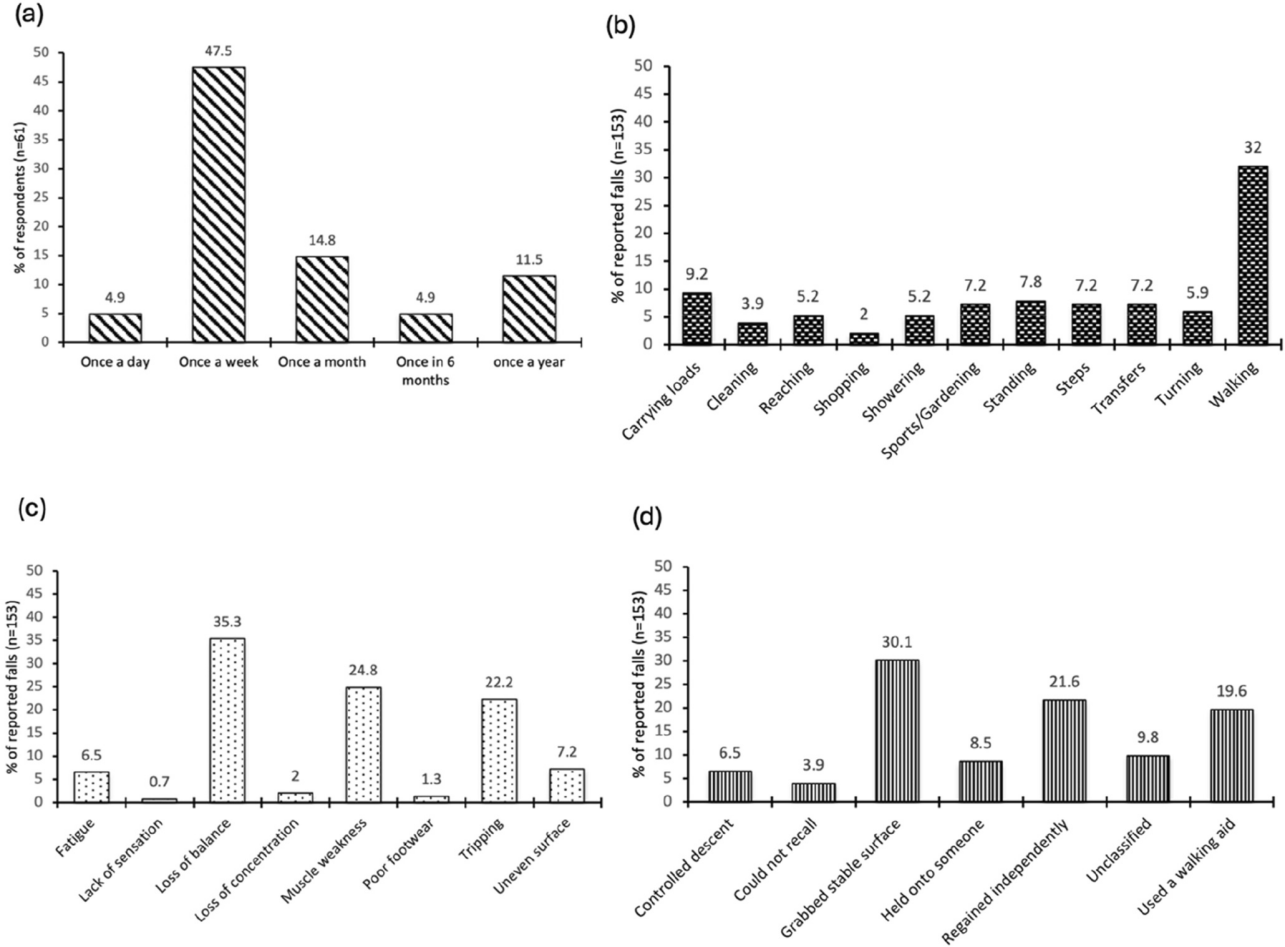
(a) Participant‐reported frequency of near‐falls in the previous 12 months (participant level; *n* = 61); (b) activity at the time of near‐fall, (c) perceived cause of near‐fall and (d) reported strategy that prevented a fall across 153 near‐fall events contributed by 51 participants (event level; up to three events per participant).

All 61 participants completed the near‐falls section, of whom 51 (83.6%) reported at least one near‐fall in the past year. Near‐falls were most often reported as occurring at least weekly (47.5%), with smaller proportions reporting monthly (14.8%), annually (11.5%), six‐monthly (4.9%) or daily (4.9%) events (Figure 2). Of the non‐fallers (*n* = 6), 0 (0.0%) reported at least 1 near‐fall.

### 3.4 Circumstances of Near‐Falls

Among the 51 participants who reported near‐falls, 153 near‐fall events were described in detail (up to three events per participant). The following findings therefore describe events rather than individual participants. Walking was the most frequent activity at the time of a near‐fall (32.0%), followed by carrying loads (9.2%), standing (7.8%), negotiating steps (7.2%), transfers (7.2%) and sports/gardening (7.2%), with further reports during turning (5.9%), reaching (5.2%), showering (5.2%), cleaning (3.9%) and shopping (2.0%). Participants most often attributed near‐falls to loss of balance (35.3%), muscle weakness (24.8%) and tripping (22.2%), with smaller contributions from uneven surfaces (7.2%), fatigue (6.5%), loss of concentration (2.0%), poor footwear (1.3%) and lack of sensation (0.7%). Participants commonly reported effective on‐the‐spot strategies to avert a fall, including grabbing a stable surface (30.1%), regaining balance independently (21.6%) and using a walking aid (19.6%); other strategies included holding onto another person (8.5%) or controlling descent (6.5%), with 9.8% unclassified and 3.9% unable to recall.

### 3.5 Exploratory Comparisons by Fall Status and Fall Frequency

All participants provided age data (fallers *n* = 55; non‐fallers *n* = 6). In this exploratory comparison, fallers were older on average than non‐fallers (mean 61.2 [SD 10.2] vs. 47.8 [SD 6.6] years; Welch's *t* = 4.39, df = 9.8, *p* = 0.002). This comparison should be interpreted cautiously given the small number of non‐fallers. Among fallers, 42 were classified as frequent (≥ monthly) and 13 as infrequent (< monthly); mean age was 62.1 (SD 9.6) and 58.2 (SD 12.1) years, respectively. At participant level, among the up‐to‐three described fall events, 0/42 frequent fallers had no injured events, 17/42 had one, 22/42 had two and 3/42 had three injured events. Among infrequent fallers, 1/13 had no injured events, 10/13 had one, 2/13 had two and none had three. Thus, 42/42 frequent fallers (100%) and 12/13 infrequent fallers (92.3%) had at least one injury recorded among their described events. These participant‐level figures relate only to the limited number of retrospectively recalled fall events described in the questionnaire and do not represent ascertainment of injuries across all falls experienced during the preceding 12 months.

## 4 Discussion

This cross‐sectional survey found that falls and near‐falls were frequently reported among adults with SBMA, with over two‐thirds of participants classified as frequent fallers. At the event level, the 153 reported fall events contributed by 55 participants predominantly occurred indoors and during walking or other functional mobility tasks, and injuries were commonly reported. Participants most frequently attributed events to lower‐limb weakness and balance loss. These perceived causes are clinically plausible given the characteristic distribution of weakness in SBMA, but the present study did not objectively determine the biomechanical or physiological mechanisms underlying individual falls.

The findings from this SBMA cohort add to growing evidence on falls in neuromuscular conditions, while highlighting condition‐specific features. As in slowly progressive disorders such as facioscapulohumeral muscular dystrophy (FSHD) (Bugiardini et al. 2022) and Charcot–Marie–Tooth disease (CMT) (Ramdharry et al. 2018), participants reported frequent falls and near‐falls during routine ambulation and functional tasks. Muscular weakness, particularly of the lower limbs, was the most commonly cited perceived contributor to falls in our survey, consistent with reports in FSHD, IBM and myotonic dystrophy (DM1) where lower limb weakness has been linked to increased fall risk and instability during stance and gait (Berends et al. 2019; Hiscock et al. 2014; Horlings, Munneke, et al. 2009). Distal weakness, including foot drop, may also contribute to tripping, although proximal and distal weakness were not differentiated in this survey (Horlings, Küng, et al. 2009).

Interestingly, very few participants in our SBMA cohort explicitly attributed their falls to sensory changes, despite the converging clinical, neurophysiological and pathological evidence that indicates SBMA includes a primary large‐fibre sensory neuropathy which could undermine proprioceptive feedback necessary for postural stability (Manzano et al. 2018). A similar pattern was noted in CMT, where only a minority of respondents identified sensory loss as a contributing factor (Ramdharry et al. 2018). This may reflect limited awareness of sensory deficits or the subtlety of sensory changes in everyday function. Quantitative testing in SBMA has demonstrated greater eyes‐closed postural sway with reduced sensory nerve input and mostly normal vestibular function, supporting a potential somatosensory/proprioceptive contribution to postural instability. Incorporating objective proprioceptive and sensory assessments in prospective SBMA cohorts would clarify how these impairments relate to day‐to‐day falls (Anagnostou et al. 2019).

Tripping and sudden balance loss were commonly reported in our cohort, often without obvious environmental triggers. These reports may be compatible with difficulties in dynamic balance control or reactive postural strategies although these mechanisms were not directly assessed in the present study. Similar mechanisms have been implicated in ALS, where slowed motor responses correlate with greater fall risk (Schell et al. 2019), and in DM1, where dynamic balance impairment is closely linked to distal lower‐limb weakness, particularly ankle dorsiflexors (e.g., step‐quick‐turn and obstacle tasks) (Bélair et al. 2022). In SBMA, Shrader et al. (2021) reported poorer performance on forward lunge, step‐quick‐turn, and step‐up‐and‐over, where many participants turned more slowly and required multiple steps, with task success related to knee‐extensor and plantar‐flexor strength. A separate study evaluating quiet stance and reactive postural control in SBMA reported greater sway with eyes closed and delayed recovery responses to perturbations (Shrader, Sansare, Niemic, et al. 2025). Taken together, these observations align with participants' reports and suggest testable hypotheses for targeted assessment and intervention.

In the present study, most falls occurred indoors, often during walking, turning, or transitional movements. This pattern mirrors findings in DM1, CMT and IBM, where the majority of falls were also reported to occur inside the home environment (Berends et al. 2019; Ramdharry et al. 2018; Hiscock et al. 2014). However, unlike studies which highlighted environmental hazards as common causes of falls, our SBMA cohort rarely identified extrinsic factors (Horlings, Munneke, et al. 2009). Instead, participants most frequently attributed their falls to intrinsic or movement‐related factors, including lower‐limb weakness, loss of balance and tripping. Notably, only a small number explicitly cited fatigue as a precipitant, despite cohort studies showing fatigue in ∼97% of men with SBMA (Alqahtani et al. 2023), and evidence that fatigue is associated with gait disturbance in other neuromuscular cohorts (Montes et al. 2011). This mismatch suggests that overall symptom burden and event‐level attribution may diverge. Environmental factors were rarely reported, suggesting that participants more commonly perceived falls as related to disease‐associated or movement‐related factors. Whether this reflects actual fall causation or under‐recognition of environmental risks requires further study.

Despite the high frequency of falls reported by participants, only three fracture events were reported. This is noteworthy in light of evidence that men with SBMA may have compromised bone health (Pradat et al. 2020). The low fracture rate may reflect fall mechanics, underreporting or compensatory behaviour, but this was not assessed.

Although formal psychosocial measures were not collected, the pattern of responses suggests active adaptation to perceived fall risk. Near‐falls were reported by the vast majority of participants, and the most common prevention strategies were grabbing a stable surface, regaining balance independently and using a walking aid. These responses suggest substantial on‐the‐spot compensatory behaviour. Fear of falling and activity modification have been reported in other neuromuscular conditions, raising the possibility of similar behavioural adaptations in SBMA (Horlings, Munneke, et al. 2009; Anderson et al. 2025). Prospective studies using measures of balance confidence and activity avoidance could examine these adaptations further.

## 5 Study Limitations

This study presents descriptive findings from a moderate‐sized cohort of individuals with genetically confirmed SBMA. Response bias is possible, as individuals with more significant fall histories or concerns may have been more likely to participate, potentially overestimating fall prevalence and severity and underrepresenting less affected individuals.

The survey relied on retrospective self‐report over the preceding 12 months, including detailed descriptions of up to three remembered falls and near‐falls. No prospective records, medical records, caregiver reports or other external sources were used to verify reported events. Consequently, fall frequency, circumstances, perceived causes and injuries may have been affected by inaccurate or selective recall. Cognitive or memory status was not formally assessed, and the ability to self‐complete the questionnaire cannot be taken as evidence that retrospective recall accuracy was established. Some fields were incomplete, and 153 of potential 165 fall events were fully described. Participants could also contribute up to three events of each type; these event‐level data therefore comprise repeated observations and should be interpreted descriptively rather than as independent observations.

Perceived causes were based on participant interpretation and may reflect multiple interacting contributors. There was no objective confirmation of impairments such as foot drop or sensory loss, and balance performance was not directly measured. Falls were not examined in relation to disease duration, SBMAFRS/mSBMAFRS, 6MWT or other objective functional measures. The study was therefore designed to describe fall frequency and circumstances rather than identify clinical predictors or mechanisms. Future studies should combine prospective fall monitoring with clinical and objective balance assessments.

## 6 Conclusion

Individuals with SBMA report a high frequency of falls and near‐falls, most often indoors during routine ambulation. Participants chiefly attributed these events to factors such as lower‐limb weakness and loss of balance, with tripping frequently described. However, disease duration, SBMAFRS/mSBMAFRS, 6MWT and objective measures of strength, balance and other functional impairments were not available. Therefore, these findings should not be interpreted as identifying clinical predictors or objectively established mechanisms of falls. Prospective studies incorporating these measures are needed to clarify motor, sensory and reactive postural contributors and evaluate potential mechanism‐based interventions.

## 7 Implications for Physiotherapy Practice

Falls and near‐falls should be routinely discussed with people living with SBMA, as they were frequently reported in this cohort and often occurred during everyday mobility tasks. Assessment may include exploration of event circumstances, perceived causes, walking‐aid use, lower‐limb strength, balance, fatigue and recovery strategies.

Potential management considerations include strengthening, gait and turning practice, dynamic balance activities, walking‐aid optimisation and strategies for higher‐risk activities. These approaches were not evaluated in this study, and their effectiveness for reducing falls in SBMA remains to be established. Given known sensory involvement in SBMA, possible proprioceptive and sensory contributions may also warrant assessment despite being infrequently reported by participants.

## Funding

This work was supported by Kennedy's Disease UK (KDUK) and by 2 year postdoctoral salary support to Laurence E. Lee. The funders had no role in study design, data collection, analysis, interpretation, manuscript preparation, or the decision to submit for publication

## Ethics Statement

Ethical approval for this study was granted by the North West—Haydock Research Ethics Committee (REC reference: 24/NW/0218; Protocol number: 1; IRAS Project ID: 335156). Approval was granted on 5 August 2024.

## Consent

All participants provided informed consent prior to taking part in the study.

## Conflicts of Interest

The authors declare no conflicts of interest.

## Data Availability

The data that support the findings of this study are available on request from the corresponding author. The data are not publicly available due to privacy or ethical restrictions.
